# Limitations of eDNA analysis for *Carcinus maenas* abundance estimations

**DOI:** 10.1186/s12862-022-01969-z

**Published:** 2022-02-07

**Authors:** Ariella M. Danziger, Zachary H. Olson, Markus Frederich

**Affiliations:** 1 School of Marine and Environmental Programs, University of New England, 11 Hills Beach Rd, Biddeford, ME USA; 2 University of New England, School of Social and Behavioral Sciences, Animal Behavior Program, 11 Hills Beach Rd, Biddeford, ME USA

**Keywords:** Environmental DNA, Biomass, Invasive species monitoring, Green crab, *Carcinus maenas*

## Abstract

**Background:**

Environmental DNA (eDNA) is an effective tool for the detection and monitoring of presence or absence of rare and invasive species. These techniques have been extended to quantify biomass in vertebrates, particularly in fish species. However, the efficacy of eDNA techniques to quantify biomass in invertebrate species has rarely been examined. This study tested whether eDNA could be used to determine the biomass of the world-wide invasive green crab, *Carcinus maena*s. In a controlled laboratory study, the relationship between biomass and *C. maenas* eDNA concentration was examined in the context of different biotic (activity) and abiotic (temperature) parameters.

**Results:**

When incubating different numbers of crabs in sterile saltwater for up to 7 days, a relationship between eDNA concentration and biomass was observed at temperatures of 6.7 ℃ and 18.7 ℃, but not at 12.8 ℃. Additionally, motor activity, aggression level, time of sampling, and features of organismal decay had significant impact on the concentration of *C. maenas* eDNA collected.

**Conclusions:**

We show that eDNA concentration did not correlate with biomass, and that biomass, temperature, organismal characteristics, and potentially many more parameters affect shedding and degradation rates for eDNA in this species, thus, impacting the recoverable eDNA concentration. Therefore, eDNA techniques are not likely to provide a reliable signal of biomass in the invasive invertebrate species *C. maenas.*

**Supplementary Information:**

The online version contains supplementary material available at 10.1186/s12862-022-01969-z.

## Background

Environmental DNA (eDNA) is frequently used to detect the presence or absence of species, particularly those of interest due to conservation status, rarity, or ecological importance [1, 2]. eDNA is any DNA released by an organism, in membrane-bound forms as individual cells or organelles, or as extracellular DNA, that can be isolated from environmental samples such as water, soil, or air [1–4]. DNA is naturally released, or shed, by organisms due to movement, through excretory processes, by advection of surrounding water, or after an organism’s death due to processes of decay [5, 6]. The use of eDNA in species survey applications is supported by the fact that eDNA degrades in the environment allowing for eDNA detections to be linked to species presence at meaningful temporal and geographic scales [4, 7–10]. However, degradations rates seem to vary greatly in freshwater and marine environments, as shown for a broad range of species, like common carp 4.2 days [8], Amphibian larvae 7–14 days [7], sturgeon, 14 days [11], bullfrog < 1–54 days [4], flounder 0.9 days [2], anchovy, sardines and mackerel 3–4 days [10], or stickleback 6.7 days [7].

The fate of eDNA, and therefore its detection and quantification, is affected by several factors that collectively have been referred to as the ecology of eDNA [12, 13], which accounts for the processes of organismal shedding of DNA, degradation rates of eDNA, and transport of eDNA within the environmental matrix. These processes are affected by hydrology (for eDNA sampling conducted in water), biology and ecology of the organism, stress levels (i.e., causing metabolic changes and thus differences in rate of release of eDNA), abiotic factors, and other biotic factors [2, 14, 15]. For example, hydrological conditions (e.g., current, wind, depth) at the site of water collections can influence transport and retention of eDNA [13, 16, 17]. The shedding and degradation rates of eDNA, as well as transport of this DNA to other locations, are affected by the biology of the organism, including its behavior, movement (both within and away from the site), individual and group characteristics, and biomass [10, 11, 18]. For example, a higher density or biomass of the species could lead to increased shedding of DNA, and thus a higher concentration of eDNA at the sampling site [10]. Lastly, the abiotic and biotic environment plays a role in all four of the factors influencing the fate of eDNA. Some examples of abiotic factors which affect eDNA include UV light, temperature, and salinity, while other biotic factors include enzyme or microbial-induced degradation [4, 16, 19, 20].

As features of the ecology of eDNA have been examined for specific systems, the utility of eDNA methods has been extended beyond presence/absence sampling to estimation of biomass or relative abundance of species, with most such efforts focusing on vertebrates [15, 21–24]. Some early eDNA studies connected species biomass with amplification rates, where higher rates of DNA amplification from species-specific assays were correlated with higher species abundances at the sample sites [e.g., 25, 26]. Quantification of eDNA from samples led to successful correlations of eDNA concentrations with species biomass, for example in laboratory studies of the common carp, *Cyprinus carpio* [22] and brook char, *Salvelinus fontinalis* [23]. However, variation in those factors affecting the ecology of eDNA in field settings have been shown to add complexity to the relationship between species abundance or biomass and eDNA concentrations [22, 23].

One promising application of eDNA methods is the detection of invasive species [27–29]. Whereas most such studies have focused on vertebrates, the use of eDNA methods has been attempted on invertebrate species, especially in more recent years (*Orconectes rustic *and *Pacifastacus leniusculus)* [30, 31]. The European green crab, *Carcinus maenas*, is an invasive invertebrate that has spread world-wide, caused damage to coastal environments, reduced marine species richness through outcompeting and consuming native species [32, 33], and destroyed eelgrass beds while feeding [34]. This destruction leads to a loss of protective habitat for a multitude of species, particularly during larval or juvenile stages [34, 35]. In the nineteenth century, *C. maenas* was introduced to the northern Atlantic Coast of the USA, and has since spread and invaded South Africa, Japan, Pacific Coast of USA, Canada, Tasmania, and Argentina [36–38] and is expected to invade Antarctica [39]. Its adaptability to new environments and competition with native organisms makes it imperative to create a reliable way to track this species’ global spread to allow for faster eradication of the species in new areas. The presence or absence of *C. maenas* has been determined using eDNA analysis in the Gulf of Maine [40], but it is unclear whether this tool can be used to determine the community biomass of *C. maenas*. Therefore, the goal of this study is to test whether the biomass of *C. maenas* correlates to the concentration of *C. maenas* eDNA in a controlled lab environment, and furthermore to test how specific features of the ecology of eDNA, abiotic and other biotic factors, alter the correlation between *C. maenas* eDNA concentrations and biomass. For *C. maenas* in particular, determining the biomass or population size in a particular area could help to adjust the methods of eradication of the species, while also determining if eradication is possible due to species abundance. Furthermore, estimating the abundance of this species could aid in predictions of changes in native populations, and thus methods of conservation of these other organisms.

## Results

### Correlation of biomass and temperature to eDNA concentration

**Fig. 1:**
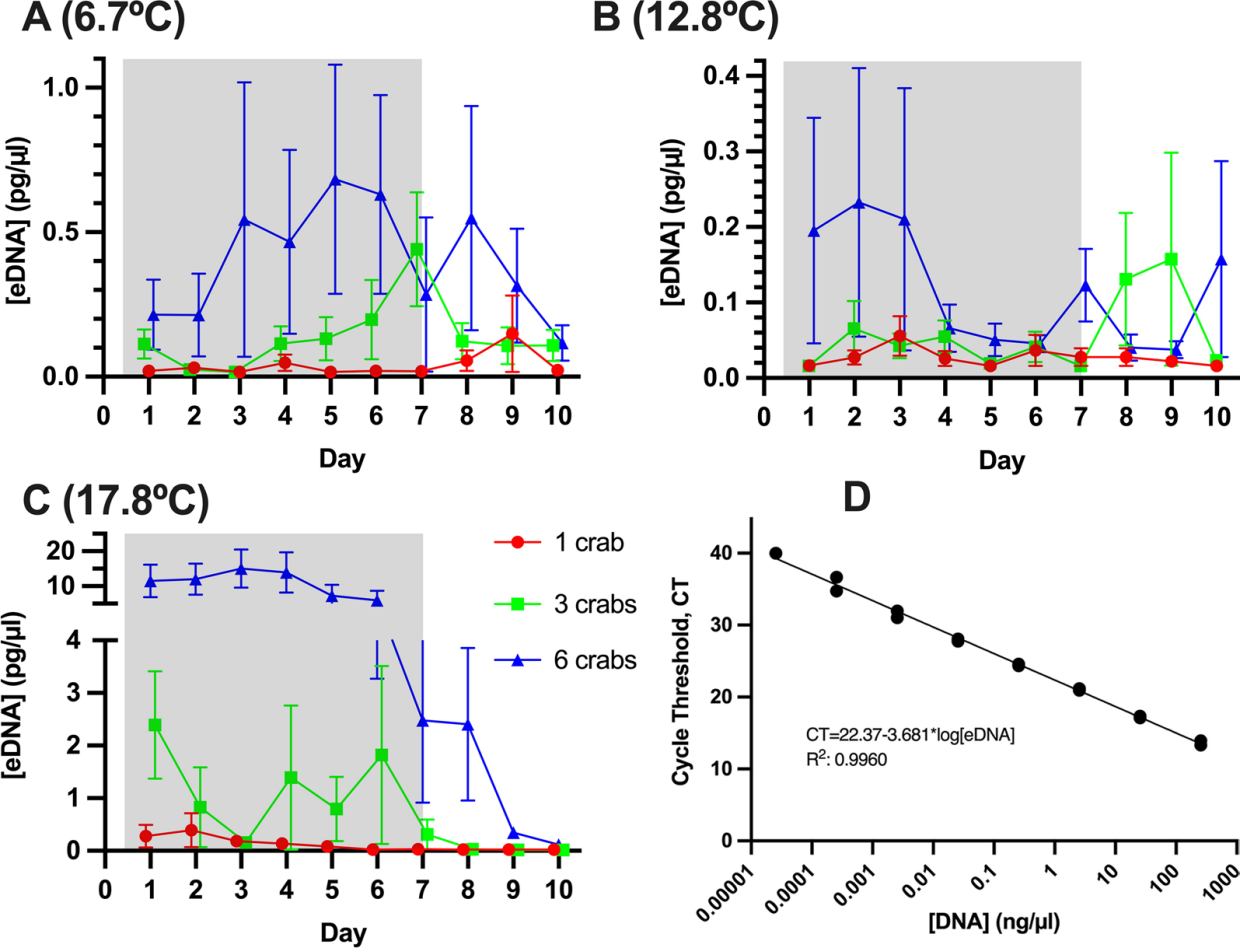
eDNA recovered over 10 days of daily sampling for 3 different densities of *C. maenas* (1, 3, or 6 crabs) within 3 separate temperatures: **A** 6.7 ℃, **B** 12.8 ℃, **C** 17.8 ℃. The grey shade within each graph represents when crabs were present in the containers, crabs were removed on day 7. Mean ± SEM, n = 5 per data point. Data points are nudged slightly along the x-axis to avoid overlaying of the respective symbols. The fourth panel (**D**) shows a representative standard curve that was used to quantify the data

The correlation between crab biomass (1, 3, or 6 crabs per bucket; 1.48 g/L, 4.3 g/L or 8.2 g/L) and crab eDNA concentration (measured by qPCR) was inconsistent. At a density of 6 crabs, eDNA concentrations differed significantly among temperature treatments (ART: F_4,352_ = 11.05, p = 1.9E−08). At 7 ℃ and 18 ℃ the largest biomass (8.2 g/L) consistently showed the highest eDNA concentration (Tukey-Test: p < 0.05). The lowest biomass revealed at multiple, but not all, timepoints the lowest eDNA concentration (Fig. 1). At 13 ℃, however, no significant differences in eDNA from the separate crab densities were found (Tukey-Test: p > 0.05). eDNA degradation after removal of the crabs from the buckets also varied with temperature. At 6.7 ℃ and 12.8 ℃ eDNA concentrations remained relatively constant. However, at 17.8 ℃ the eDNA concentration quickly decreased, presumably due to DNA degradation (ART: F_18,352_ = 3.32, p = 1.4E−08).

### Behavior

#### Activity

**Fig. 2:**
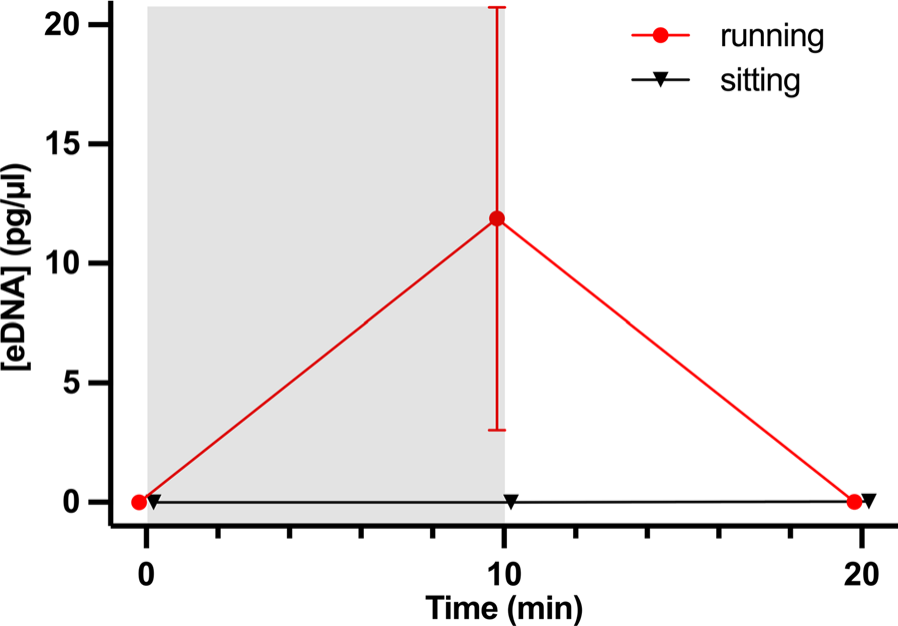
eDNA recovered from crabs walking on an underwater treadmill for 10 min and subsequent rest of 10 min, compared to 20 min rest only. Active running on the motor-driven belt by the active crab is represented by the grey shade. Mean ± SEM, n = 5 per data point. Data points are nudged slightly along the x-axis to avoid overlaying of the respective symbols

After 10 min of running on an underwater treadmill at about 60% of their maximum speed, the active crabs released a significantly higher amount eDNA compared to the stationary crabs, which were immobile on the treadmill (ART: F_1,24_ = 16.75, p = 4.17E−4; Fig. 2). Stationary crab eDNA was not different from control values (p > 0.05). After 10 min of running and a resting period of 10 min (time point 20 min), eDNA concentration in the active condition decreased to low concentrations no different from that of the stationary crab (0.0199 ± 0.014 vs. 0.0278 ± 0.023 pg/µl, respectively; p > 0.05).

#### Aggression

**Fig. 3:**
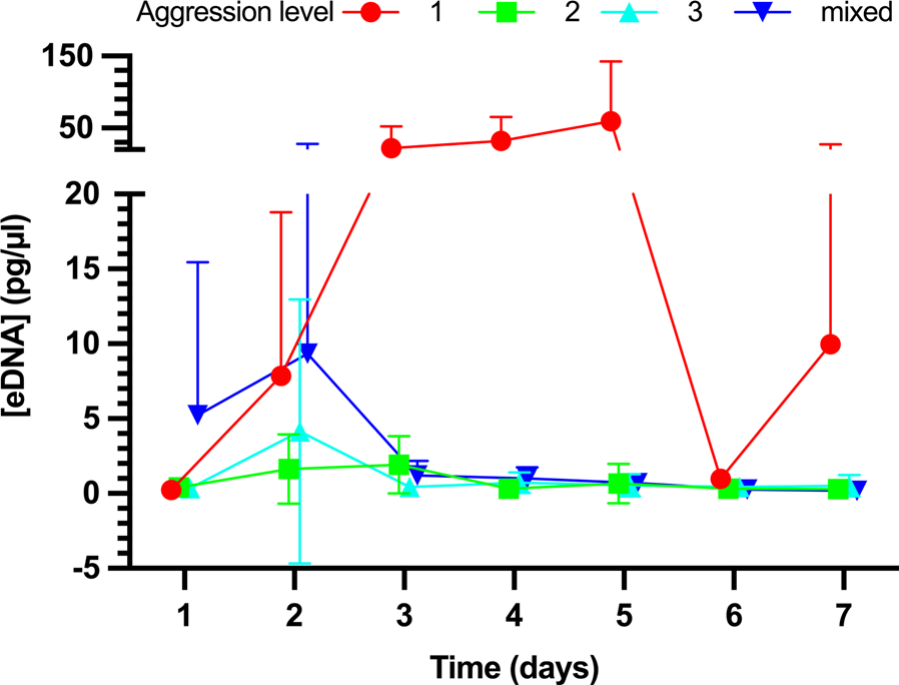
eDNA release over seven consecutive days for 3 separate aggression levels of *C. maenas*, and all 3 combined levels of aggression (mixed). Mean ± SEM, error bars are shown in only one direction for some data points for clarity of the figure, n = 5 per data point. Data points are nudged slightly along the x-axis to avoid overlaying of the respective symbols

Crabs with different aggression levels exuded variable amounts of eDNA over time (Fig. 3). Consistently, however, aggression level 1 crabs (low aggression) released significantly more eDNA than the crabs of aggression level 2, 3, or that of the mixed aggression groups (ART: F_4,112_ = 12.7, p = 1.5E−08).

### Organismal decay

**Fig. 4:**
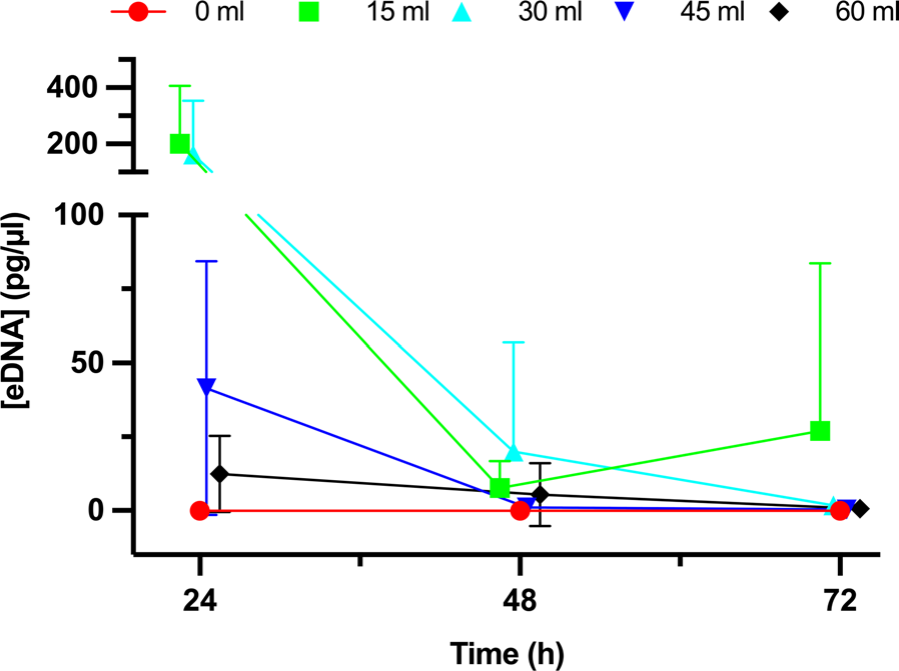
eDNA released of collected eDNA samples at 3 time points for 72 h after addition of 0 mL, 15 mL, 30 mL, and 45 mL of crab stock solution to 15-L of sterile seawater. Mean ± SEM, error bars are shown in only one direction for some data points for clarity of the figure. N = 5 per data point. Data points are nudged slightly along the x-axis to avoid overlaying of the respective symbols. Though not visible due to scale, one qPCR replicate from the 0 mL treatment yielded a false positive amplification

Lower amounts of solution from decaying (homogenized) crabs yielded a higher eDNA concentration (F_4,51_ = 6.9, p = 1.5E−05) (Fig. 4). Furthermore, time of sampling had a significant effect on eDNA concentration; after 24 h, the amount of eDNA collected significantly decreased (ART: F_2,51_ = 13.8, p = 1.6 E−05).

### eDNA recovery

**Fig. 5:**
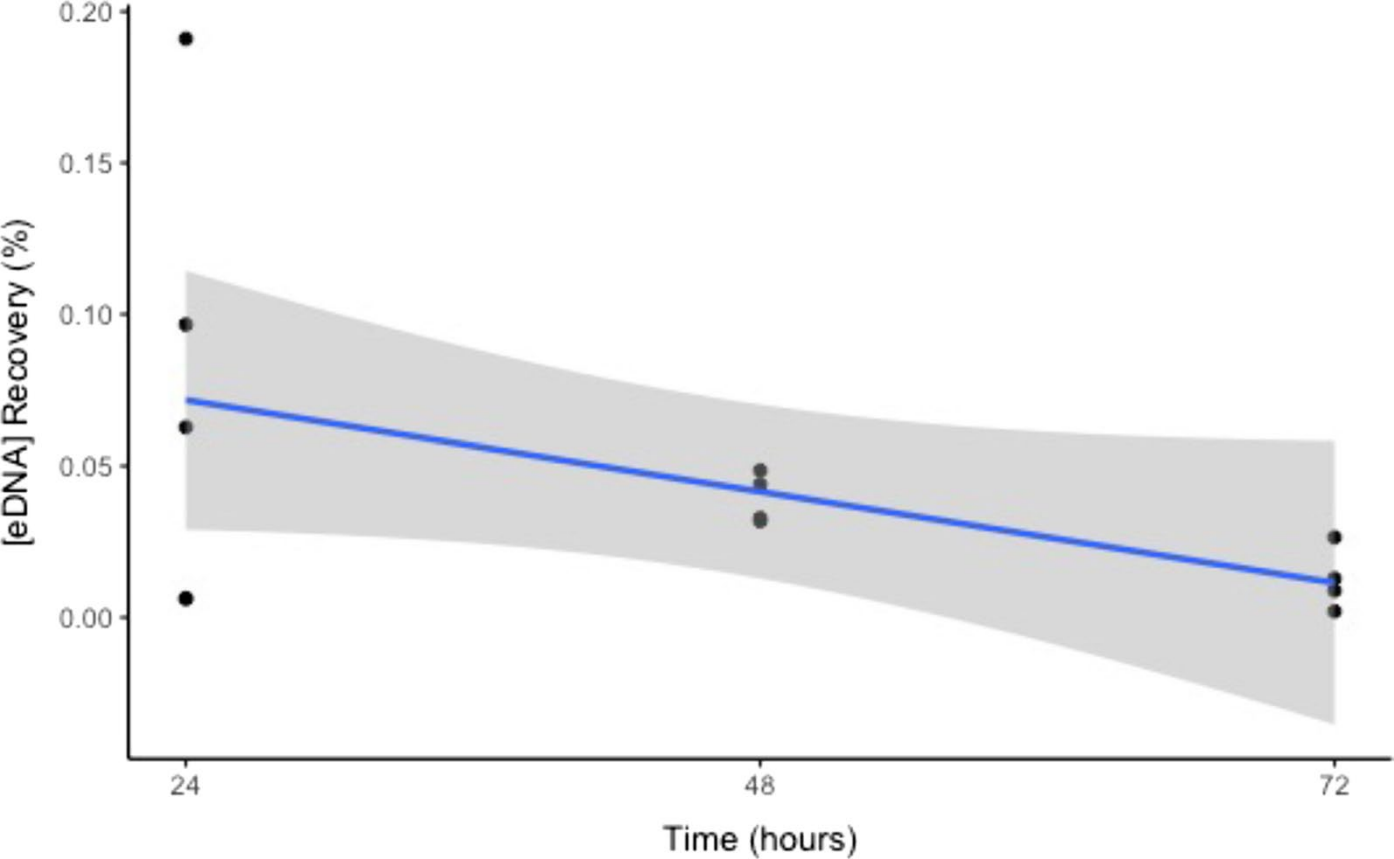
[eDNA] recovery percentage over three time points within 72 h. The solid line represents the regression line (R^2^ = 0.996), and the grey shaded area represents the standard error of the regression line

The percentage of [eDNA] recovery continuously decreased over a 72-h period and exhibited a negative linear relationship with time (y = -0.001255x + 0.101783, R^2^ = 0.996, p = 0.0225) (Fig. 5).

### Water filtration

**Fig. 6:**
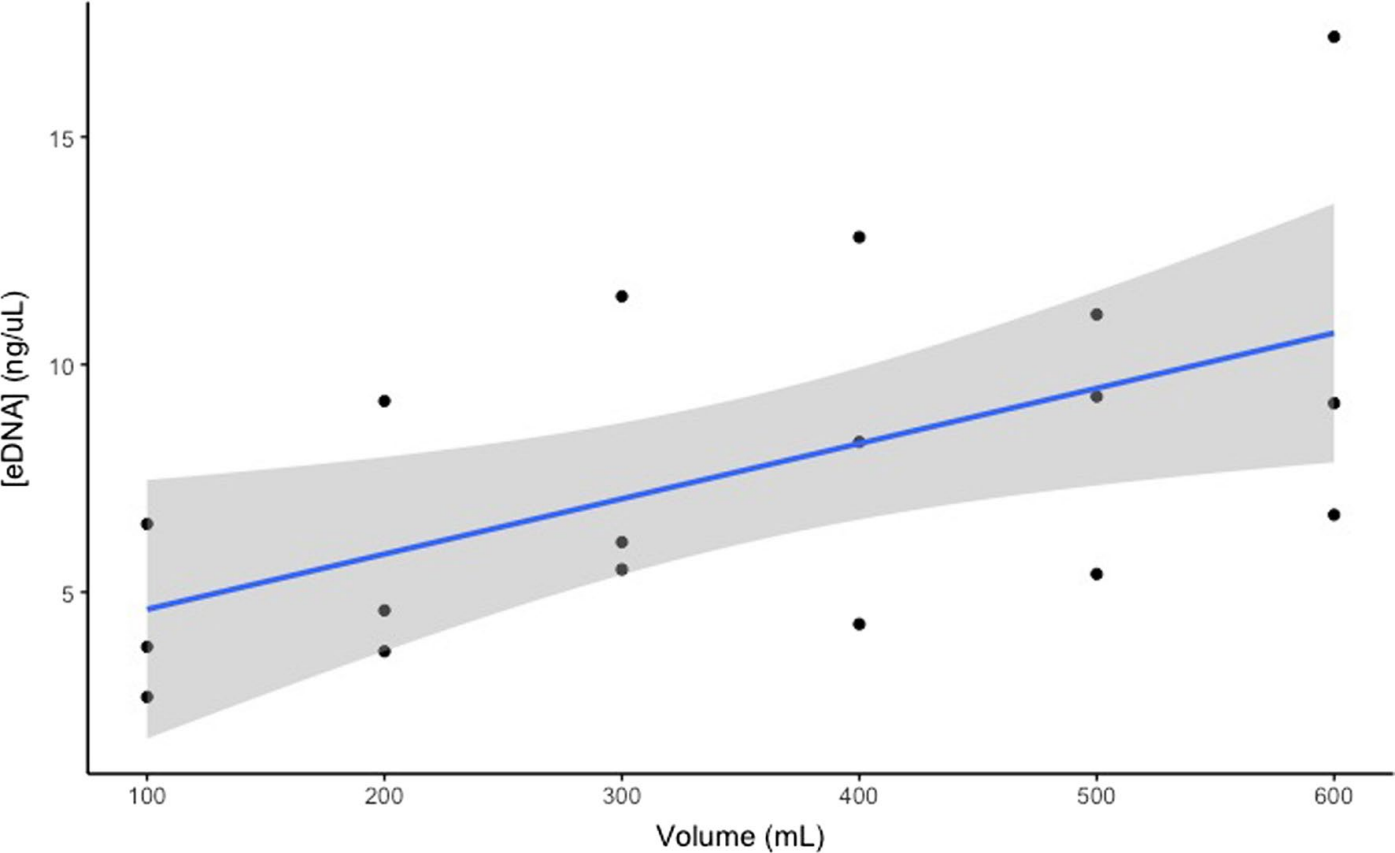
DNA concentration from six volumes of filtered water from 100 to 600 mL. The solid line represents the linear regression line (R^2^ = 0.95) and the grey shaded area represents the standard error of the regression line

A linear relationship (y = -0.0107x + 38.2829, R^2^ = 0.95, p = 0.0225) existed between 100 and 600 ml filtered water and the respective recovered eDNA concentration (Fig. 6). Volumes of 100 ml were close to the detection limit, volumes of 400 mL and larger easily clogged the filters. As a result, the smallest volume of water we could filter and continuously get DNA amplification with was 200 ml. All water was subsequently sampled and filtered as 200 ml.

### Contamination

The control buckets held for all experiments with only sterile artificially made seawater consistently had no amplification of DNA.

## Discussion

The first part of our study examined the relationship between *C. maenas* eDNA concentration and *C. maenas* density (1, 3, and 6 crabs), and how this relationship is impacted by seawater temperatures (6.7 ℃, 12.8 ℃, and 17.8 ℃), the range of which was chosen to reflect changes in the yearly average water temperature observed in the Gulf of Maine [41, 42]. Our results demonstrate that eDNA concentration cannot consistently be related to biomass of *C. maenas*. While a density of 6 crabs produced eDNA concentrations that were different from those of 1 and 3 crab treatments, this difference was only observed at two temperatures (6.7˚C and 17.8 ℃). Work by Lacoursiére-Roussel et al. [22] on brook char at two different temperatures (7 ℃ and 14 ℃) revealed that shedding rates scale proportionally with temperature, where an increase in temperature led to a higher shedding rate of eDNA. This could be due to changes in mobility and the metabolic rate of organisms at higher temperatures [9, 18, 22, 43]. Our study shows that eDNA analysis cannot be used to rigorously predict biomass of *C. maenas* in a controlled lab experiment and suggests against eDNA methods for that purpose in the field.

Multiple factors, aside from temperature, could influence the relationship (or lack thereof) between *C. maenas* biomass and eDNA concentration. General shedding rates of eDNA depend on a multitude of factors including temperature, stress, diet, biomass, life stage, and individual organismal characteristics [4, 10, 18, 22, 43].

First, we tested motor activity for its effect on *C. maenas* eDNA release. As expected, increased crab activity triggered greater release of eDNA compared to immobile crabs. The rapid decrease of an eDNA signal just 10 min after ending the walking activity was very surprising, but highly reproducible in the experiment. Further tests will need to investigate why the eDNA signal disappeared that rapidly but persisted much longer in the other bucket experiments. We tested the effect of motor activity on eDNA release only at one temperature (7.8 ℃), but it is likely that temperature-induced activity levels contribute to increased eDNA release rates at increasing temperatures. As an ectotherm, responding to temperature changes with a metabolic rate following a Q_10_ of 2–3, *C. maenas* increases its activity at higher water temperatures [44]. In a separate experiment, we found that at 17.8 ℃, active crabs released significantly more eDNA compared to active crabs in the two lower temperature treatments. Thus, it can be expected that crabs at this higher temperature released more eDNA at least partially due to increased, temperature-induced activity, as suspected in the experiment listed above.

In the same context, we also investigated the effect of aggression level on eDNA release. We predicted that high aggression crabs would release the most eDNA but found that the lowest aggression crabs released the most eDNA. Reasons for these unexpected results have not been investigated further. However, future studies should determine if activity level amongst aggression levels differs. For example, if level 1 (low aggression) crabs avoid higher level aggression crabs by evasion, and higher-level aggression crabs stand their ground, level 1 crabs would have more motor activity and therefore released more eDNA. Besides testing eDNA released between aggression levels, scenarios in which all aggression levels of crabs were mixed together were also tested. The amount of eDNA detected was similar to that of medium and high aggression. Furthermore, eDNA levels seemed to oscillate over time in all aggression groups. It is possible that overall activity levels varied over time, leading to variation in eDNA release. The consistency and reproducibility of this oscillating eDNA release suggests a specific process is involved. However, identifying this process was not possible without monitoring or controlling for the voluntary motor activity of the crabs over time, which was not a focus of the current study. Nevertheless, our results suggest that aggression levels impact the amount of eDNA being released in an environment. Notably, aggression levels do vary among populations. Recent results show that in a subsample of more than 300 crabs *C. maenas* from Maine, 58% were aggression level 1, 42% were level 2, and 0% were aggression level 3, while crabs from Nova Scotia were 30% level 1, 50% level 2, and 20% level 3 aggression [45]. Given these findings, if eDNA correlations to biomass were made, it would be necessary to take into account not only the seawater temperature and motor activity, but also the percentage of each aggression level within the population in question.

eDNA is not only released from live animals, but also from dead ones, including those that were fed on by predators or conspecifics and therefore potentially released large amounts of eDNA [3]. If more crabs are present one can expect more mortalities, thereby impacting the amount of eDNA in the water column. Our simulations of organismal decay (i.e., by adding different amounts of homogenized crab to water), produced unexpected results: samples with smaller volumes of homogenized crab resulted in higher recoverable eDNA concentrations than did higher volume treatments. These results might be explained by larger volumes of homogenized crabs also containing more nucleases and other DNA degrading enzymes, potentially leading to greater DNA degradation or PCR inhibition [8, 46]. Such results clearly demonstrate that the simple notion or prediction of more crab biomass (in this case dead) leading to more eDNA is not consistent or correct.

The three factors that we found affecting eDNA concentrations were activity level, temperature, and biomass (at 6.7 ℃ and 17.8 ℃). Activity level specifically has not previously been studied for its impact on eDNA concentration. However, while working with juvenile and adult bluegill sunfish, *Lepomis macrochirus,* Maruyama et al. [18] found that juveniles released higher amounts of eDNA, possibly due to their higher activity and metabolic rate. Similarly, our study demonstrated that eDNA concentration increased with higher motor activity of *C. maenas*. Furthermore, Crane et al. [47] found that larval stage affects eDNA release in *C. maenas*. While their study focused on eDNA from sediment samples, the study concluded that ovigerous organisms shed more DNA than those in other life stages, though eDNA concentration and detection was still low [47].

Temperature can delay or accelerate DNA degradation. Colder temperatures have been shown to delay degradation, while warmer water temperatures speed degradation [4]; eDNA degradation rates at 5 ℃ are much slower compared to those at 20 ℃ or higher [4, 9, 16]. The increased degradation rate at higher temperatures is due to several factors. Increased microbial and enzymatic activity in warmer temperatures can stimulate DNA degradation [20, 48, 49]. Lower temperatures also slow microbial DNA decay, thus aiding to the slowed overall eDNA degradation rate [9, 50]. Our experiments show that with higher temperatures, more eDNA is released, however degradation also appears to occur faster in both our experiment and the published data.

Multiple studies in fish species have shown that biomass is positively correlated with eDNA concentration. Klymus et al. [15] found a linear relationship between eDNA concentration and fish biomass for both bighead carp and silver carp. A similar study by Takahara et al. [23] confirmed this linear relationship in common carp. By contrast, others have found no relationship between species’ abundance and eDNA concentration in *Cryptobranchus alleganiensis* (hellbender salamander) [51], *Triturus cristatus* (northern crested newt) [52], *Orconectes rusticus* (rusty crayfish) [30, 31], *Pacifcastacus leniusculus* (American signal crayfish) [31]. The differences in relationships between eDNA and species biomass could be due to the multitude of confounding variables, such as those which affect shedding and degradation rates, timing of sampling throughout the life cycle or breeding season, and many more [51]. The findings of our study align with those examining other crustaceans [30, 31]. For *C. maenas,* while eDNA concentration does increase with biomass at some temperatures, biomass determinations could not be consistently made using this technique.

The effect of organismal decay on eDNA release and detection has scarcely been studied. Our data show that as the amount of decomposing organismal matter increases, the eDNA detected decreases. Possible inhibition of PCR due to enzymatic activity released during decay was investigated, and no inhibition of primers was occurring. Such findings are similar to examinations by Curtis and Larson [53] of invasive red swamp crayfish (*Procambarus clarkia*). In a swamp enclosure, the carcasses of this crayfish released no detectable eDNA into the water column. Similar to our study, inhibition of primers was tested for, and no inhibition was found. Thus, this study suggested that carcasses may not release a detectable amount of eDNA, suggesting that live organisms contribute most, if not all, eDNA detected.

Time of sampling was the last factor analyzed within this study. As time since release of eDNA into the water column increases, the amount of eDNA degradation increases, however this rate of degradation can vary between species and amount of eDNA released [4, 16]. For this reason, time of sampling and the subsequent eDNA detected was analyzed using *C. maenas*. Our results were congruent with previous data, i.e., that of the eDNA detection of the Idaho giant salamander, showing that time of sampling after introduction and removal of a species to a water source adjusts the amount of eDNA detected. After the introduction to a new location, the collected eDNA of the Idaho giant salamander (*Dicamptodon aterrimus*) exhibited an initial high release of eDNA. After removal of the species, eDNA concentrations found in the water samples began to degrade and was undetectable after 3 days with *D. aterrimus* [16]. Similar to our study with *C. maenas*, DNA quickly began to degrade, however detection after 3 days depended on the temperature of the water.

## Conclusions

**Fig. 7:**
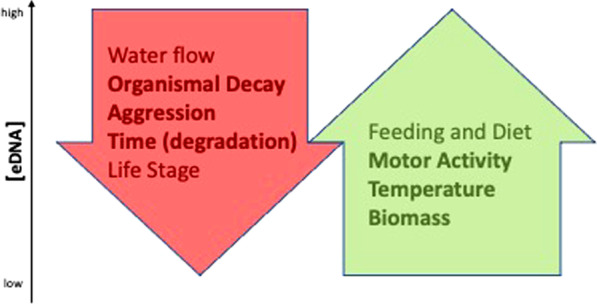
Observed and anticipated correlations between multiple abiotic and biotic factors (feeding and diet, motor activity, temperature, biomass, water flow, organismal decay, aggression, time, and life stage) and eDNA concentration detected in the water column. Factors examined in this study are bolded (motor activity, temperature, biomass, organismal decay, aggression, and time).

Overall, our study shows that various abiotic and biotic parameters alter the concentration of *C. maenas* eDNA in the water, via increasing or decreasing shedding and degradation rates. If individual parameters affecting eDNA concentration could be separated, a clear correlation between eDNA concentration and the respective parameter might be possible. Our conceptual model (Fig. 7) illustrates potential correlations between parameters investigated in this study and those reported in the literature with at least two competing trends in two different directions. For determining biomass of a population of animals in the water at different temperatures, depths, and directions of flow, where the animals show different activity levels, aggression levels, life stages, feeding status, and many other not controlled conditions (for example, we did not measure pH levels throughout our incubations and pH can have an effect on eDNA release and recovery; [4] and citations therein), the resulting eDNA concentration is merely a sum of all these effects. In the case of *C. maenas*, a high eDNA concentration could be generated by a few highly active crabs present at the time of the water sampling, which are feasting on several dead crabs. By contrast, the same high eDNA concentration might have come from many inactive crabs having left the area some time ago, but an initially higher eDNA level produced by these inactive crabs had decreased by degradation. A multitude of scenarios are possible, and the current biotic and abiotic scenario present at the time of eDNA sampling is generally unknown. Therefore, a determination of biomass of *C. maenas*, and potentially of many other species, is likely to be inaccurate when using eDNA. Our study, along with previous findings [30, 31] demonstrate that more studies should be conducted to determine exactly which species’ biomass sizes can be determined using eDNA quantification.

## Methods

### Experimental design

Crabs for this study were collected by hand in the rocky intertidal zone on the oceanic side of Biddeford Pool, Maine (43.442292 °N, 70.341244 °W) and kept in a 500-L tank in a sea water flow-through system in the Marine Science Center of the University of New England. Additional male *C. maenas* were hand collected and trapped in Kejimkujik Seaside National Park, Nova Scotia, Canada (43.50323 °N 64.50041 °W). These were transported in coolers by car to the Marine Science Center of the University of New England in Biddeford, Maine, where they were held in separate 300 L tanks with a flow through sea water system. Water discharged from the system was filtered through 1 µm filters and sterilized using 2 UV filters (QL-40 Lifegard Ultraviolet Sterilizer) before release into the local river. As a secondary containment, all of this was housed in a 4-m diameter pool. The setup was permitted and inspected by the Maine Department of Marine Resources (permit numbers S2013-007, S2014-007, S2015-008, S2016-008, S2017-007). Animals were fed frozen fish and squid ad libitum. Institutional Animal Care and Use Committee approval is not required for experiments involving invertebrates.

In a controlled lab experiment we incubated different numbers of green crabs (*C. maenas*) under different conditions (biomass, motor activity, organismal activity, aggression level, temperature, and eDNA degradation; details are outlined below). For all laboratory experiments, artificial seawater (Instant Ocean SeaSalt with deionized water, 32 ppt) was used to avoid DNA contamination from other sources. Water was stored in a 120-L container enclosed in a wooden box to aid in the exclusion of further DNA contamination. Furthermore, water was treated with UV light for 10 min before use to ensure sterilization of water.

Fifteen liters of artificial sea water were placed in sterile 19-L buckets containing bleached bubble stones connected to an air pump. A lid over this bucket minimized the DNA contamination from the laboratory. These 19-L containers were used for the different treatments described below. As a negative control, a bucket with only sterile sea water was continuously tested for eDNA in parallel to all experiments.

### Correlation of biomass and temperature to eDNA concentration

Crabs were selected based on size and weight. Weight, carapace width, sex, and abdominal color were recorded for each crab. Crabs were grouped into densities of 1, 3, or 6 per bucket, equating to 22.2 ± 3.0, 64.4 ± 3.8, and 123.4 ± 9.1 g respectively. Density groupings were kept at 6.7 ℃, 12.8 ℃, or 17.8 ℃ for 7 days. Temperatures were chosen to correspond to seasonal water temperatures in the Gulf of Maine. Every 24 h, 200 mL of water was collected from each bucket and filtered as described below. The removed water was not replaced with new water to keep the potential for contaminations minimal, therefore the overall volume of water decreased slightly throughout the incubations. To minimize this decrease in water we determined the minimum amount of filtered water that provides reproducible data (200 mL), see Sections “Water filtration” for details. After 7 days, crabs were removed from the buckets and water was sampled daily for an additional 3 days to measure degradation rates. All treatments (densities of *C. maenas* within various temperatures) and sample collections were replicated 5 times.

### Behavior

#### Motor activity

We compared eDNA release between when *C. maenas* were resting and running on an underwater treadmill. The treadmill consisted of an acrylic aquarium and a motorized belt (30 × 20 cm) and perforated box (20 × 19 × 14 cm; L × W × H) to keep the crabs centered on the treadmill [54]. Before use, the treadmill was sterilized with 10% bleach and rinsed with deionized water. It was then filled with 15-L sterile artificial seawater, and 200 mL of this water was collected for filtration. Crabs ran on the belt for 10 min at a speed of 20 cm/s (about 60% of their maximum running speed, as determined prior to the experiments), and then rested for 10 min. Water was agitated and samples were taken after 10 min running and 10 min resting. Size-matched crabs rested on the belt for 20 min as controls with the same water collection time points. All water samples were filtered immediately. The treadmill was sterilized between each trial. We tested 10 crabs in total, 5 running, 5 at rest, thus taking 5 replicates of each treatment in this experiment.

#### Aggression

Aggression levels of green crabs were ranked from 1 (least aggressive) to 3 (most aggressive). When agitated with a probe touching their face crabs with a level 1 aggression kept their claws close to their body and ran away. Level 2 aggression crabs opened their claws into a meral spread and held their stance. Level 3 aggression crabs attacked the probe and other nearby crabs immediately.

Crabs were chosen haphazardly, and their aggression levels determined. Crabs from the Gulf of Maine had level 1 or 2 (low or medium) aggressions only. Therefore, we also used crabs from Nova Scotia, which showed consistently higher aggression levels (2 or 3) [55]. Crabs were paired in 19-L buckets as follows: 3 crabs level 1, 3 crabs level 2, 2 crabs level 3 (level 3 crabs from Nova Scotia were larger; to maintain similar biomass, only 2 were used), and 2 crabs level 1 with 2 crabs level 2 and 1 crab level 3. The total biomass per bucket for aggressions 1, 2, and 3 was 112.9 ± 9.9 g, the mixed aggression bucket was 224.3 ± 8.3 g. Every 24 h for one week 200 mL of water were collected from each bucket. Water collected was immediately filtered. Five trials of this experiment were completed.

### Organismal decay

To simulate organismal decay, 4 crabs (biomass average of 32 g) were crushed and then homogenized with 400 mL water using a blender to create a stock solution. This solution was mixed into buckets with 15-L of sterile sea water in four volumes: 15, 30, 45, and 60 mL (n = 5 replicate buckets per volume of stock solution). Water collection for filtration occurred every 24 h for 3 days. 200 mL of water was immediately filtered after collection. After qPCR analysis, it was found that samples expected to have high eDNA concentrations in fact showed the opposite trend. Thus, we tested to ensure PCR inhibition did not occur. We spiked a sample of DNA isolated from the crab homogenate with DNA isolated from crab hepatopancreas. Comparing the respective PCR results of DNA from the hepatopancreas alone, from the crab homogenate, and the crab homogenate spiked with DNA from the hepatopancreas showed predicted DNA concentrations and therefore no PCR inhibition by the crab homogenate.

### eDNA recovery

DNA from the hepatopancreas of *C. maenas* was isolated using the Qiagen DNeasy Blood and Tissue Kit following the protocol for DNA isolations from tissue. The concentration of this DNA was quantified using a NanoDrop™ 2000 Spectrophotometer. 50 µL of 255 ng/µL DNA was added to 15-L of sterile seawater (n = 5 replicates). 200 mL of water was filtered every 24 h for three days. Percent recovery was determined using the total genomic DNA concentration as the starting point of our recovery.

### Water filtration

A water filtration system with four 300 mL filtration funnels and sterile 0.45 µm cellulose nitrate filters (Sartorius, Germany; #11,306–47-ACN) connected to a vacuum pump (Gast DOA-P7004-AA) was constructed (see Additional file 1: Fig. S1). In a lightproof box a 7 W UV light was used to sterilize the filtration setup for 10 min before each water filtration. After water samples were filtered, filters were stored in individual sterile 2 mL Eppendorf tubes at −80 ℃ until DNA isolation.

For determinations on the minimum volume of water that could be collected and filtered to test for the presence of eDNA from *C. maenas*, water was sampled from a 500 L tank containing more than 100 green crabs in six volumes (100, 200, 300, 400, 500, and 600 mL, n = 3 per volume). This water was filtered and eDNA was isolated using the Qiagen DNeasy Blood and Tissue Kit. DNA was amplified using the designed primers and TaqMan probe sequence for qPCR, and total DNA concentration was measured using a ThermoFisher NanoDrop™ spectrophotometer.

### DNA isolation and quantitative PCR

The Qiagen DNeasy Blood and Tissue Kit was used to isolate DNA from filters following the manufacturer’s instructions with minor modifications. 180 µL of buffer ATL and 20 µL proteinase K were added to the filter and incubated at 56 ℃ for 10 min. 200 µL of buffer AL was then added to the samples and incubated for another 10 min at 56 ℃. After all incubations, 200 µL of ethanol was added and samples were centrifuged for 1 min at 8000 rpm to aid in the separation of the liquid from the filter. The liquid was pipetted into a DNeasy Mini Spin column and centrifuged once more for 1 min at 8000 rpm. The column was washed with 500 µL buffer AW1 and 500 µL of buffer AW2. 200 µL of buffer AE was used to elute the DNA from the spin column and eluted DNA was stored in −80 ℃.

Quantitative real-time PCR (qPCR) was performed on isolated DNA samples in duplicates with primers and TaqMan probe sequences specific to *C. maenas* in the Gulf of Maine [40]. Sequences were forward primer 5’-AAT ATT GGG AGG GCC AGA TAT AG-3’, reverse primer 5’-AGG ATC GAA GAA TGA GGT GTT TAG-3’, TaqMan probe 5’- 6-FAM-GGT TCT GAT TAC TTC CTC CGT CTT TAA CCT-MGB -3’. qPCR conditions were 15 min at 95 ℃; then 40 cycles of 15 s at 95 ℃ and 1 min at 62 ℃. The last segment was one cycle for 1 min at 95 ℃, 30 s at 62 ℃, and 30 s at 95 ℃. For every qPCR run, two positive and negative (blank) controls were run along with the collected samples. Samples with a cycle threshold (CT) below 40 were considered positive amplification. Samples that did not reach the CT after 40 cycles were considered negative, and we used the value of 0 (zero) to calculate the respective average concentrations.

To quantify the DNA concentration with qPCR, DNA was isolated from the hepatopancreas of *C. maenas* using the Qiagen DNeasy Blood and Tissue Kit and the concentration was determined using a NanoDrop™ 2000 Spectrophotometer. A standard curve of CT and a dilution series of eDNA concentrations between 255 and 2.55 × 10^–5^ ng/µl was created (Fig. 1D) and used quantification. The R^2^ for this standard curve was 0.996, the slope of −3.681 represents an efficiency of 82.7%

### Statistics

For all experiments, one or two-way type III ANOVAs were used to test for the effects of each variable on the cycle threshold (CT) or eDNA concentration. One-way ANOVAs were used for water eDNA recovery and water filtration; two-way ANOVAs were used for biomass and temperature, activity, aggression, and organismal decay. P-values less than 0.05 were considered significant. Assumptions of normality and heteroscedasticity were checked with Shapiro-Wilks and Levene tests, respectively. For experiments with only one variable (motor activity and eDNA recovery) Kruskal–Wallis tests were performed. Data with more than one variable were analyzed with the Aligned Rank Transform (ART) if assumptions for ANOVA were not met [56]. If the statistical test was significant, we used post-hoc Tukey tests to differentiate significant treatments. Data are shown as means ± 1SEM.

## Supplementary Information

**Additional file 1: Figure S1. **Photo of the filtration setup. Four filter funnels are housed in a light proof plywood box that allows for UV sterilization prior to filtration. 2 water traps collect the water and protect the vacuum pump.

## Data Availability

The datasets generated during and/or analyzed during the current study are available from the corresponding author on request.

## Abbreviations

CT: Cycle Threshold
eDNA: Environmental DNA
qPCR: Quantitative real-time polymerase chain reaction
