# Supplementary material for: Limitations of eDNA analysis for Carcinus maenas abundance estimations
Source: BMC Ecol Evol. 2022 Feb 7;22:14. doi: 10.1186/s12862-022-01969-z (PMC8822865; doi:10.1186/s12862-022-01969-z)
Supplement: Supplementary file 1 — Additional file 1: Figure S1. Photo of the filtration setup. Four filter funnels are housed in a light proof plywood box that allows for UV sterilization prior to filtration. 2 water traps collect the water and protect the vacuum pump. [file 12862_2022_1969_MOESM1_ESM.docx]

Supplemental material


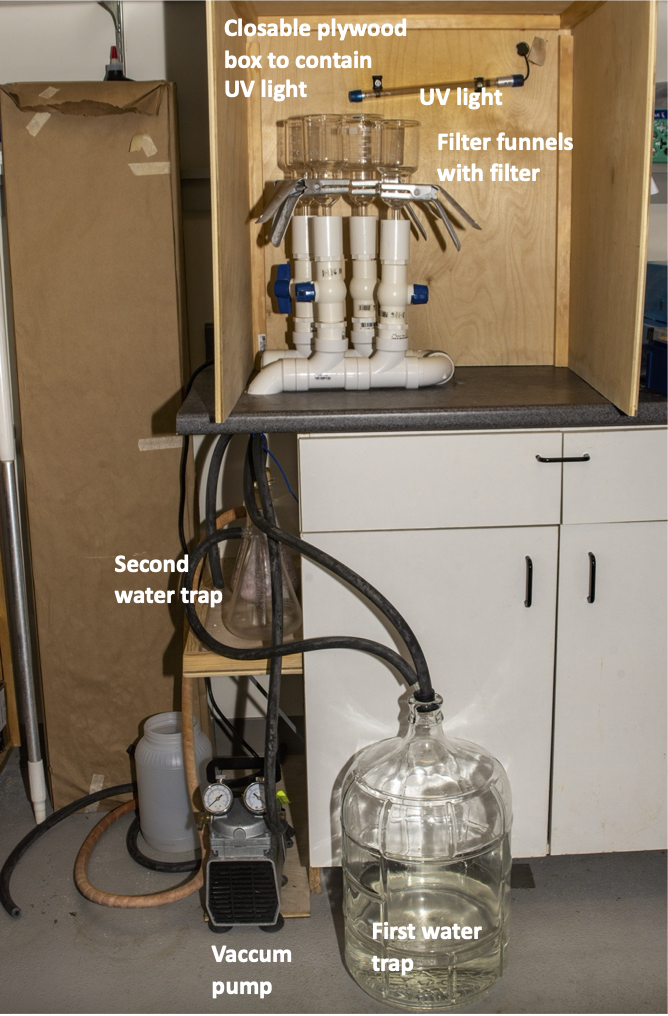


Figure S1:

Photo of the filtration setup. Four filter funnels are housed in a light proof plywood box that allows for UV sterilization prior to filtration. 2 water traps collect the water and protect the vacuum pump.
